# Immune recovery in acute and chronic HIV infection and the impact of thymic stromal lymphopoietin

**DOI:** 10.1186/s12879-016-1930-3

**Published:** 2016-10-21

**Authors:** Marco Gelpi, Hans J. Hartling, Kristina Thorsteinsson, Jan Gerstoft, Henrik Ullum, Susanne D. Nielsen

**Affiliations:** 1 Department of Infectious Diseases, Viro-Immunology Research Unit, University Hospital of Copenhagen Rigshospitalet, Copenhagen, Denmark; 2 Department of Infectious Disease, University Hospital of Copenhagen Hvidovre, Copenhagen, Denmark; 3 Department of Clinical Immunology, University Hospital of Copenhagen Rigshospitalet, Copenhagen, Denmark

**Keywords:** TSLP, HIV, Primary HIV infection, Chronic HIV infection

## Abstract

**Background:**

Symptomatic primary HIV infection is associated with an adverse prognosis, and immediate initiation of combination antiretroviral therapy (cART) is recommended. However, little is known about immunological predictors of immune recovery. Thymic Stromal Lymphopoietin (TSLP) is a cytokine that promotes CD4+ T cells homeostatic polyclonal proliferation and regulates Th17/regulatory T-cell balance, immunological functions known to be affected during primary HIV infection. The aim of this study was to describe immune recovery in primary and chronic HIV infection and possible impact of TSLP.

**Methods:**

Prospective study including 100 HIV-infected individuals (primary HIV infection (*N* = 14), early presenters (>350 CD4+ T cells/μL, *N* = 42), late presenters without advanced disease (200–350 CD4+ T cells/μL, *N* = 24) and with advanced disease (<200 CD4+ T cells/μL, *N* = 20) and). Immune recovery was defined as increase in CD4+ T cells count from baseline to a given time of follow-up. Plasma TSLP was determined using ELISA and CD4+ T cell subpopulations (recent thymic emigrants, naïve and memory cells) were measured using flow cytometry at baseline and after 6, 12 and 24 months of cART.

**Results:**

Immune recovery was comparable in all groups, and no differences in immune homeostasis were found between primary HIV infection and early presenters, whereas differences in absolute counts and proportions of CD4+ T cell subpopulations were found between primary HIV infection and late presenters. TSLP was elevated in primary HIV infection at baseline and after 24 months of cART. Interestingly, TSLP was negatively associated with proportion of recent thymic emigrants (correlation coefficient −0.60, *p* = 0.030). TSLP was not associated with immune recovery in primary HIV infection.

**Conclusions:**

Immune recovery was comparable in primary and chronic HIV infection whereas differences in absolute counts and proportions of CD4+ T cell subpopulations were found between primary HIV infection and late presenters supporting early initiation of cART. Higher plasma TSLP was found in primary HIV infection, and TSLP was associated with lower thymic output, but not with immune recovery. These findings indicate a possible role of TSLP in immune homeostasis in HIV infection but do not support TSLP to affect immune recovery in primary HIV infection.

## Background

WHO estimates that there are two million new cases of HIV infection each year. It is estimated that 40-90 % of newly infected individuals have symptomatic acute primary HIV infection, a mononucleosis-like syndrome that appears right after the infection [1–3]. An association between symptomatic acute primary infection and a worse prognosis in terms of decline in CD4+ T cells count and progression to AIDS has been described [4–6]. Initiation of combination antiretroviral therapy (cART) during the primary HIV infection has been shown to result in faster recovery of CD4+ T cells when compared to initiation of cART during chronic phase of infection [7–9]. Noteworthy, HIV-infected individuals that maintain a CD4+ T cell count above 500 cells/μL have been shown to have lower risk of both AIDS and all cause mortality [10]. Furthermore, beneficial effect of early cART on both chronic immune activation and viral reservoir has been described [11, 12]. However, little is known about the immunological predictors of immune recovery in individuals with primary HIV infection, and only few studies have addressed T cell subsets distribution in primary and chronic HIV infection [13, 14].

Our group has recently shown that Interleukin-7 (IL-7) and IL-7 receptor (IL-7R) plasma concentrations are lower in chronic untreated HIV infection, especially in those individuals with a low CD4+ T cell counts. Interestingly, previous studies have demonstrated both high plasma IL-7 and low T cell expression of IL-7R to be associated with suboptimal CD4+ T cell recovery [15–19]. Furthermore, a high IL-7 induced T cell response has been shown to be associated with higher CD4+ T cell counts [20] suggesting an important role for IL-7 and IL-7R in CD4+ T cell recovery in HIV infection. Thymic Stromal Lymphopoietin (TSLP) and IL-7 are closely related cytokines both with a prominent role in CD4+ T cell homeostasis [21]. TSLP is produced by epithelial cells in skin, lung, gut, and thymus and the TSLP receptor (TSLPR), a heterodimer composed of CD127 and cytokine receptor-like factor 2 (CRLF2), is widely distributed on CD4+ T cells and dendritic cells (DC) reflecting a wide range of actions [22]. TSLP has mainly been linked to priming of naïve CD4+ T cell towards a Th2 response [23]. Interestingly, TSLP educated DC have been shown to have an important role in the homeostatic polyclonal expansion of naïve CD4+ T cells [24]. Even more interesting, TSLP produced by the Hassal’s corpuscles in the thymic medulla has been shown to affect thymic output of CD4+ T cell in healthy HIV-negative individuals [25]. However, the impact of TSLP on T cell subset distribution during primary and chronic HIV infection has not previously been reported.

This study was conducted to describe immune recovery in primary and chronic HIV infection. Furthermore, we aimed to determine circulating TSLP in primary and chronic HIV infection and the impact of TSLP on immune recovery. We hypothesized that immunological impairment would be less pronounced and immune recovery faster in individuals with primary HIV infection compared to individuals with chronic infection with a comparable baseline CD4+ T cell count. Finally, we hypothesized that TSLP would be associated with immune recovery. Thus, total CD4+ T cell counts as well as proportions and absolute numbers of recent thymic emigrants (RTE), naïve and memory CD4 + T cells as well as plasma concentrations of TSLP were measured in a total of 100 individuals with either primary or chronic HIV infection prior to initiation of cART and after 6, 12 and 24 months.

## Methods

### Study population

Plasma samples were obtained from 100 treatment naïve HIV-infected individuals as previously described [26]. Inclusion criteria were a positive HIV-1 test and initiation of cART. Decision about cART initiation was made by the patient’s physician and the patient, and participation in this study did not influence this decision. All individuals were enrolled from Department of Infectious Diseases, Rigshospitalet, Copenhagen University Hospital or Department of Infectious Diseases, Hvidovre Hospital. A total of 14 participants were defined as having primary HIV infection (PHI), i.e. they had a negative HIV test less than 6 months prior to diagnosis and/or one or more negative bands in Immune Blot. Fiebig stages for patients with PHI are shown in Table 1. Participants with chronic HIV infection were further divided in three subgroups, according to CD4+ T cell count at baseline: Late presenters with advanced disease (LP-AD, <200 CD4+ T cells/μL at presentation, *N* = 20), late presenters without advanced disease (LP-nonAD, 200–350 CD4+ T cells/μL at presentation; *N* = 24), and early presenters (EP, >350 CD4+ T cells/μL at presentation, *N* = 42). The majority of study participants were males (90.7 %), and the median age was 39.1 years (IQR: 31.4-45.2). Blood samples from 18 HIV-negative individuals were obtained as controls (Table 1). No differences in sex and age distribution were found between the groups.

**Table 1:** Clinical characteristics of the study population

	PHI	LP-AD <200 cells/μL	LP-nonAD 200–350 cells/μL	EP >350 cells/μL	HC
	*N* = 14	*N* = 20	*N* = 24	*N* = 42	*N* = 18
Gender, males/females, (% males)	13/1 (92.9)	18/2 (90.0)	21/3 (87.5)	39/3 (92.9)	17/1 (94.4)
Age, years, median (IQR)	47 (12)	42 (16)	38 (16)	44.5 (12)	42.5 (12)
Time since diagnosis, days, median (IQR)	2 (3)	3 (9)	18 (269)	24 (983)	NA
CD4+ nadir, cells/μL, median (IQR)	540 (335)	45 (113)	290 (95)	480 (170)	NA
CD4+ at baseline, cells/μL, median (IQR)	550 (327)	55 (110)	290 (97)	510 (172)	983 (540)
CD4/CD8 at baseline, median (IQR)	0.5 (0.3)	0.1 (0.1)	0.3 (0.1)	0.5 (0.3)	1.5 (0.9)
Co-infection with chronic HBV/HCV, N	0/1	0/2	0/0	1/1	0/0
HIV-RNA at baseline, median (IQR)	151,775 (3,442,296)	196,589 (751,023)	65,990 (89,637)	49,422 (47,031)	NA
AIDS defining events, N	0	1	0	0	NA
Fiebig Stage I, N	1	NA	NA	NA	NA
Fiebig Stage II, N	1	NA	NA	NA	NA
Fiebig Stage III, N	1	NA	NA	NA	NA
Fiebig Stage IV, N	11	NA	NA	NA	NA

*Abbreviations: LP-AD* late presenters with advanced disease, *LP-nonAD* late presenters without advanced disease, *EP* early presenters, *PHI* primary HIV infection, *HC* healthy controls, *NA* not applicable

Data comparing proportion of CD4+ T cell subsets in the different groups of chronic HIV infection and healthy controls is presented in another manuscript [26]. Therefore, in the present manuscript only comparisons between PHI and the different groups of chronic HIV infection and healthy controls are made.

### Collection of blood samples and flow cytometry

Blood samples from HIV-infected participants were collected at the day of inclusion (baseline/day 0 of cART) and after 6, 12 and 24 months of cART. Blood samples from healthy controls were only collected once at the time of inclusion. The numbers of samples available at each timepoint (baseline, 6, 12 and 24 months after initiation of cART) were: PHI: 14, 11, 8, 6; LP-AD: 20, 11, 11, 9; LP-nonAD: 24, 12, 14, 8; EP: 42, 24, 20, 10.

**Fig. 1:**
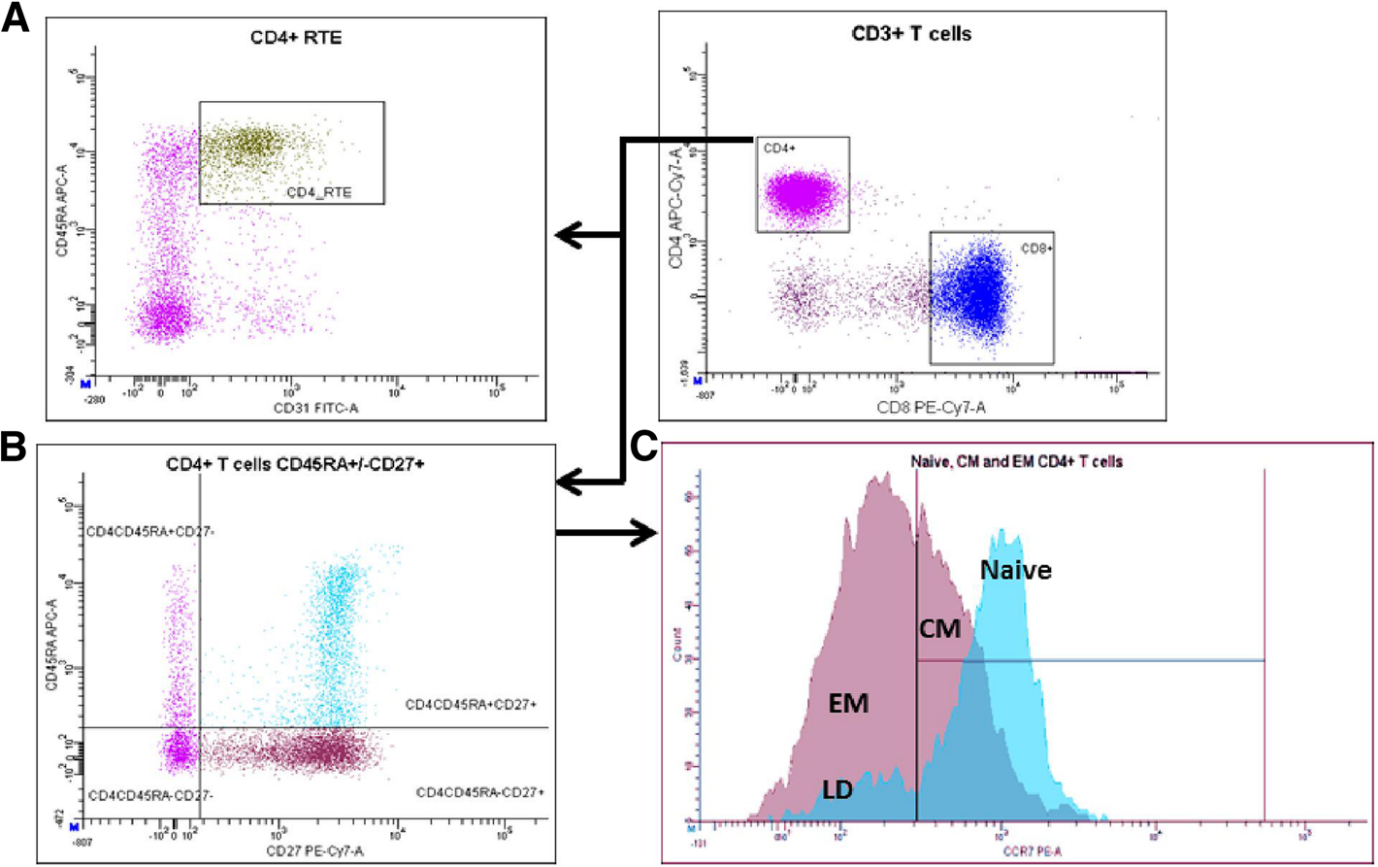
Gating strategy for CD4+ T cells subpopulations. Gating of CD4+ RTE (CD4 + CD45RA + CD31+) (**a**) Gating of naive CD4+ T cells (CD45RA + CD27 + CCR7+), central memory CD4+ T cells (CM, CD45RA-CD27 + CCR7+), late differentiated CD4+ T cells (LD, CD45RA + CD27-CCR7-), and effector memory CD4+ T cells (EM, CD45RA-CD27 + CCR7-) (**b**, **c**)

Blood collected in ethylenediamine tetraacetic acid (EDTA) tubes was used for flow cytometry as described previously [26]. In brief, 100 μL of EDTA blood was incubated with fluorescent dye–conjugated monoclonal antibodies, erythrocytes were lysed with 2 mL of Lysing Solution (Becton Dickinson (BD), Franklin Lakes, NJ, USA) and the samples were washed and resuspended in Facs flow (BD). CD3 was used in combination with CD4 to identify recent thymic emigrants (RTE, CD45RA + CD31+), naive cells (CD45RA + CD27 + CCR7+), central memory (CM, CD45RA-CD27 + CCR7+), effector memory (EM, CD45RA-CD27 + CCR7-), and late differentiated CD4+ T cells (LD, CD45RA+, CD27-, CCR7-). Monoclonal antibodies used to determine lymphocyte subsets were CD8- PE-Cy7-A, CD3-fluorescein isothiocyanate, CCR7-Phycoerythrin (PE), CD31-FITC, CD45RA-APC, CD27-PE-Cy7 and CD4-APC-Cy7-A, and appropriate isotype controls all purchased from BD. Acquisition was performed using a FACS Canto, and data were processed using FACS Diva software (BD). For each sample a minimum of 100,000 cells were acquired and gated as previously described by our group [27]. Representative plots and gating strategy is shown in Fig. 1.

### TSLP determination by enzyme linked Immunosorbent assay

The concentration of TSLP was determined in snap frozen plasma separated within 30 min after collection and immediately stored at −80 °C. ELISA kit (eBioscience, San Diego, CA, USA) was used according to manufacturer’s instructions. Plasma concentration of TSLP was determined in HIV-infected individuals with available plasma samples at baseline (*n* = 90), 6 months after initiation of cART (*n* = 57), 12 months after initiation of cART (*n* = 53), and 24 months after initiation of cART (*n* = 33). Plasma concentration of TSLP was determined in all healthy controls (*n* = 18).

### Statistical analysis

Differences between the groups of HIV-infected individuals were evaluated by Kruskal-Wallis test and if significant followed by Mann-Whitney *U* test. The effect of baseline measures of TSLP on immune recovery and changes in CD4 + T cell subsets distribution after initiating cART were evaluated by Spearman test. Immune recovery was defined as increase in CD4+ T cells count from baseline to a given time of follow-up. When exploring correlations between TSLP and CD4+ T cell subpopulations, data from patients in each group were analyzed separately. Two-tailed P-values <0.05 were considered significant. All statistical analyses were performed using SPSS (version 22 - IBM corp., Armonk, New York, USA).

## Results

### CD4+ T cell count and CD4+ T cell subsets before and after initiation of cART

Clinical characteristics of the study population are shown in Table 1.

The CD4+ T cell count was comparable in PHI and EP at all time points (Table 2). In contrast, differences in CD4+ T count between PHI and LP-AD and LP-nonAD were found at baseline (Table 2). These differences remained 6 and 12 months after initiation of cART. A difference between PHI and LP-AD was also found after 24 months of cART (Table 2). No differences in CD4+ T cell recovery between PHI and any of the groups with chronic infections at any of the follow-up time points were found.

**Table 2:** CD4+ T cell subsets proportion in primary HIV infection before and after initiation of cART

		Primary HIV (PHI)	Chronic patients CD4 < 200 (LP-AD)	Chronic patients CD4 200–350 (LP-nonAD)	Chronic patients CD4 > 350 (EP)	P*
		*N =* 14	*N =* 20	*N =* 24	*N =* 42	
Cells/μL	CD4					
	Baseline	550 (327)^a,b^	55.5 (110)^a^	290 (97)^b^	510 (172)	**< .001**
	After 6 months of cART	740 (375)^a,b^	210 (280)^a^	450 (190)^b^	690 (265)	**< .001**
	After 12 months of cART	720 (435)^a,b^	340 (170)^a^	505 (198)^b^	740 (210)	**< .001**
	After 24 months of cART	680 (240)^a^	269 (160)^a^	695 (290)	820 (317)	**< .001**
% CD4 Cells	RTE					
	Baseline	14 (11)	11 (16)	20 (15)	18 (14)	.063
	After 6 months of cART	16 (11)^b^	8 (18)	27 (15)^b^	19 (15)	**.006**
	After 12 months of cART	8 (17)	6 (20)	17 (25)	5 (25)	.588
	After 24 months of cART	18 (9)^b^	17 (10)	28 (11)^b^	17 (16)	**.009**
	Naive					
	Baseline	43 (20)^a^	23 (30)^a^	40 (26)	44 (21)	**< .001**
	After 6 months of cART	37 (23)	22 (31)	49 (19)	39 (20)	**.031**
	After 12 months of cART	46 (30)	28 (28)	49 (19)	45 (24)	.066
	After 24 months of cART	36 (12)^b^	30 (16)	55 (16)^b^	37 (15)	**.002**
	EM					
	Baseline	12 (7)	17 (14)	16 (12)	12 (6)	**.009**
	After 6 months of cART	7 (4)^a^	18 (11)^a^	6 (4)	7 (4)	**.001**
	After 12 months of cART	9 (6)^a^	15 (14)^a^	7 (5)	6 (3)	**.007**
	After 24 months of cART	9 (4)^a^	15 (6)^a^	6 (7)	7 (5)	**.043**
	CM					
	Baseline	26 (6)	20 (22)	24 (10)	24 (10)	.597
	After 6 months of cART	29 (11)	35 (18)	27 (15)	28 (14)	.969
	After 12 months of cART	26 (8)	24 (16)	24 (11)	25 (10)	.949
	After 24 months of cART	24 (14)	30 (23)	24 (8)	32 (16)	.784
	LD					
	Baseline	5 (4)^b^	3 (12)	1 (2)^b^	5 (8)	**.009**
	After 6 months of cART	8 (11)^b^	3 (8)	1 (3)^b^	5 (7)	**.012**
	After 12 months of cART	7 (14)	4 (11)	2 (3)	7 (12)	.126
	After 24 months of cART	9 (16)^a,b^	1 (1)^a^	3 (2)^b^	9 (13)	**.038**

Data are presented as median and (IQR)

*Abbreviations: LP-AD* late presenters with advanced disease, *LP-nonAD* late presenters without advanced disease, *EP* early presenters, *PHI* primary HIV infection

P*: comparing the four HIV groups by using Kruskal-Wallis test. If significant (<0.05) then Mann-Whitney was used to compare PHI group with the other chronic groups. Only significant differences are marked: ^a^PHI vs LP-AD; ^b^PHI vs LP-nonAD; ^c^PHI vs EP

Significant *P* values are shown in bold

RTE and naïve CD4+ T cells were determined as markers of thymic output, while EM, CM and LD were determined to characterize the memory CD4+ T cell subsets. No differences in proportion of these subsets were found between PHI and EP at any timepoint. In contrast, compared to LP-nonAD, PHI had lower proportion of RTE after 6 and 24 months of cART and lower proportion of naïve CD4+ T cells after 24 months of cART (Table 2). No differences in effector memory CD4+ T cells were found at any timepoint. However, PHI had higher proportion of late differentiated CD4+ T cells before and after 6 and 24 months of cART (Table 2). When comparing PHI and LP-AD no differences in proportion of RTE were found, while PHI had higher proportion of naïve CD4+ T cell at baseline (Table 2). Furthermore, PHI had lower proportions of effector memory CD4+ T cells compared to LP-AD 6, 12 and 24 months after initiation of cART. Finally, PHI had higher proportion of late differentiated CD4+ T cell after 24 months of cART compared to LP-AD (Table 2).

**Fig. 2:**
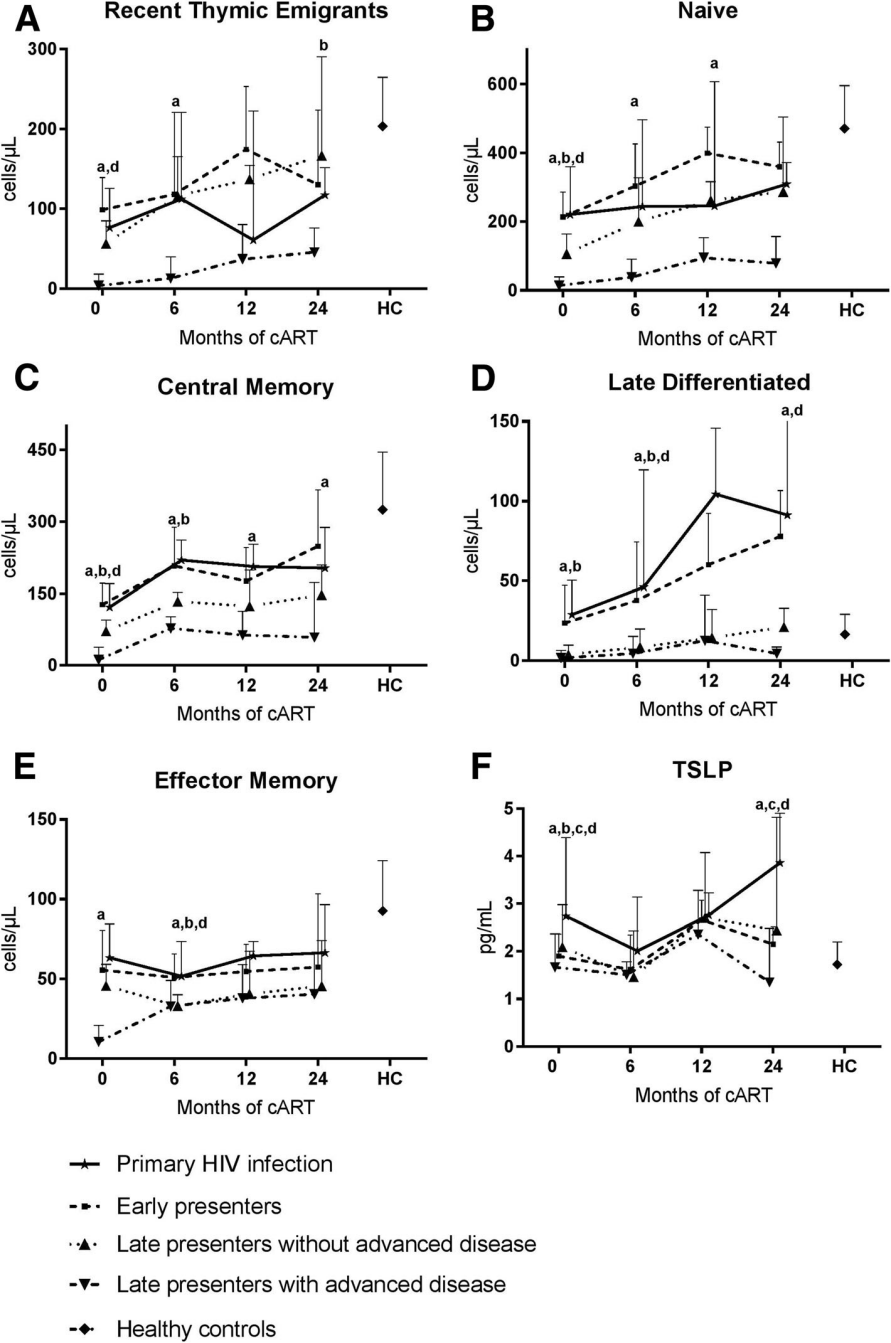
CD+ T cell subset prior to cART and during 24 months of follow up. Absolute counts of recent thymic emigrants (**a**), naïve (**b**), central memory (**c**), late differentiated (**d**) and effector memory (**e**) CD4+ T cells. Plasma concentration of TSLP (**f**). The numbers of samples available at each timepoint (baseline, 6, 12 and 24 months after initiation of cART) were PHI: 14, 11, 8, 6; LP-AD: 20, 11, 11, 9; LP-nonAD: 24, 12, 14, 8; EP: 42, 24, 20, 10. Mann-Whitney was used to compare PHI group with the other chronic groups and healthy controls. Significant differences are marked: a: PHI vs. late presenters with advanced disease; b: PHI vs. late presenters without advanced disease; c: PHI vs. early presenters; d: PHI vs. healthy controls

When comparing absolute cell counts, no differences in RTE, naïve, CM, EM or LD cell counts were found between PHI and EP at any timepoint (Fig. 2a-e). When comparing PHI and LP-nonAD RTE cell count was lower in PHI after 24 months of cART (Fig. 2a). In contrast, the baseline naïve cell count was higher in PHI than in LP-nonAD (Fig. 2b). Both CM and LD cell counts were higher in PHI compared to LP-nonAD at baseline and after 6 months of cART (Fig. 2c and d). When comparing PHI and LP-AD, the RTE cell count was higher in PHI at baseline, and 6 and 24 months after the initiation of cART. Furthermore, PHI had higher naïve and LD cell counts compared to LP-AD before cART, and after 6 and 24 months. Finally, EM and CM cell counts were higher in PHI than in LP-AD at baseline and after 6 months of cART (Fig. 2c and e), and CM cell count remained higher in PHI after 12 and 24 months.

### CD4+ T cell subsets in patients with primary HIV infection before and after initiation of cART versus healthy controls

The proportion of late differentiated CD4+ T cell was higher in PHI than in HC at baseline and after 6 and 24 months of cART, while PHI had lower proportion of RTE than HC after 12 months of cART. No differences in proportion of naïve, effector memory and central memory CD4+ T cell between PHI and HC were found (data not shown). At baseline, PHI had lower absolute count of RTE, naïve and central memory CD4+ T cells than HC (Fig. 2a, b, c). Furthermore, PHI had lower effector memory absolute count compared to healthy controls after 6 months of cART (Fig. 2e), while the absolute count of late differentiated CD4+ T cells was higher in PHI than in HC both at 6 and 12 months of follow-up (Fig. 2d).

### TSLP and IL-7 in plasma

Plasma concentrations of TSLP were determined at baseline and after 6, 12 and 24 months of cART (Fig. 2f). Prior to initiation of cART, concentration of TSLP was higher in PHI than in HC and higher than in all the groups of individuals with chronic infections (LP-AD, LP-nonAD and EP). This difference was also found between PHI and both LP-AD and EP after 24 months of cART (Fig. 2f). IL-7 was previously measured in this cohort [26]. Significant associations were not found between IL-7 and TSLP concentrations in any of the HIV-infected groups at any time points.

### Association between TSLP and CD4+ T cell count and subsets at baseline

To determine possible impact of TSLP on CD4+ T cell recovery, associations between TSLP at baseline and CD4+ T cell recovery at all time points after initiation of cART were investigated. Higher plasma TSLP was associated with lower CD4+ T cell recovery at 12 months in the LP-nonAD group (correlation coefficient −0.50, *P* = 0.034). No other associations between TSLP and CD4+ T cell recovery were found. Furthermore, the time period from initiation of cART to a CD4+ T cells count ≥ 500 cells/μL was analyzed for all participants, and significant correlation between this time period and plasma TSLP was not found in any of the HIV-infected groups (data not shown).

In addition, possible associations between TSLP at baseline and proportion of CD4+ T cell subsets at baseline were investigated. In PHI, plasma TSLP at baseline was negatively associated with proportion of RTE (correlation coefficient −0.60, *P* = 0.030). Furthermore, TSLP at baseline was associated with a higher proportion of effector memory cells in the EP group (correlation coefficient 0.33, *P* = 0.048). No other significant associations were found.

## Discussion

HIV-infected individuals presenting for care with primary HIV infection are offered immediate initiation of cART, but information about immune homeostasis in this group of individuals and possible predictors of CD4+ T cell recovery is incomplete. Both TSLP and IL-7 are cytokines with important roles in regulation of CD4+ T cell homeostasis. We and others have found plasma Interleukin-7 and Interleukin-7R to impact CD4+ T cell recovery after initiation of cART [19, 26]. In contrast, little is known about potential roles of TSLP in HIV infection. To study immune recovery and impact of TSLP in both primary and chronic HIV infection a prospective study of 100 individuals that initiated cART and were followed up for two years was conducted. Individuals with primary HIV infection and early presenters presented for care with comparable CD4+ T cell counts, and immune homeostasis and CD4+ T cell recovery in these two groups were similar. Interestingly, higher plasma TSLP was found in PHI, compared to both chronic HIV infection and healthy controls, and TSLP was associated with lower proportion of RTE in PHI. However, TSLP was not associated with CD4 + T cell recovery in this group. In contrast, TSLP was associated with lower immune recovery in LP-nonAD.

Primary and chronic HIV infection is characterized by differences in CD4+ T cell homeostasis [13, 28, 29]. However, to our knowledge, this is the largest prospective study to describe both thymic output (naïve and recent thymic emigrants) and memory cells (central and effector memory) in both acute and chronic HIV-infected individuals. Naïve CD4+ T cells are one of the first CD4+ T cell subpopulations to be infected during primary HIV infection [13]. This causes a rapid decline in CD4+ T cells which continues at a slower rate in untreated individuals during the chronic phase of HIV infection [13]. Accordingly, a higher proportion of naïve CD4+ T cells was found in PHI compared to LP-AD before the initiation of cART. Furthermore, the proportion of effector memory CD4+ T-cell is known to gradually increase during HIV infection [30, 31]. In line with these previous findings, we found lower proportion of CD4+ effector memory cells in PHI compared to LP-AD at 6, 12 and 24 months after the initiation of cART. In contrast, differences in subsets of CD4+ T cells or in CD4+ T cell immune recovery were not found when comparing PHI and early presenters that had comparable CD4+ T cell counts at baseline. Our findings suggest a beneficial effect of early initiation of cART on both proportion of naïve and effector memory CD4+ T cells. However, individuals with primary HIV infections and early presenters initiating cART early seem to have comparable T cell subsets distribution at baseline and comparable immune recovery.

TSLP is an IL-7 like cytokine mainly produced by epithelial cells in skin, lung, gut and thymus [32]. Due to its role in priming of naïve CD4+ T cells towards a Th2 response, TSLP has been described to have an essential role in the initiation of allergic diseases [33, 34]. Interestingly, TSLP has a prominent role also in promoting homeostatic polyclonal proliferation of CD4+ T cells and in regulating Th17/regulatory T-cell balance [25, 35]. All factors that are affected even in the early stages of HIV infection [11, 36, 37]. With this in mind, we speculated that TSLP would be less perturbed in PHI compared to chronic HIV infection. No previous studies of plasma TSLP in HIV infection exists. However, Fontenot and colleagues described HIV-induced TSLP mRNA expression in human genital epithelial cells in early stages of the infection [38]. We found higher plasma TSLP in individuals with primary HIV infection compared to both chronic HIV infection and healthy controls. Interestingly, individuals who initiated cART during primary HIV infection maintained higher concentration of TSLP compared to LP-AD and EP even after 24 months of cART, suggesting that timing of initiation of cART has important long-term effects on TSLP homeostasis. However, TSLP was not associated with CD4+ T cell recovery, and the impact of this finding remains unclear. Interestingly, TSLP was a negative predictor for CD4+ T cell recovery in HIV-infected individuals with a low CD4+ T cell count at the time of initiation of cART. We speculate that in individuals with impaired immune system increased TSLP may be a response to low CD4+ T cell counts rather than a cause of poor immune recovery.

Interestingly, high plasma TSLP in untreated individuals with PHI was associated with a lower proportion of RTE. We speculate that loss of CD4+ T cells during primary HIV infections leads to increased production of TSLP, but unfortunately we do not have access to samples at the time of seroconversion and therefore not data to support this hypothesis. On the other hand, plasma TSLP was not associated with a lower thymic output in any of the groups with chronic HIV infection. However, the latter finding may be due to the limited number of patients during the follow-up. TSLP has been suggested to be involved in maintaining a homeostatic polyclonal proliferation in the CD4+ T cell central memory compartment in healthy individuals [24]. In contrast, we found no correlation between TSLP plasma levels and the proportion of central memory CD4+ T cells, albeit a positive correlation between TSLP and the proportion of effector memory cells was found in early presenters. This discrepancy may be due to different responses in HIV-infected and uninfected individuals.

The present study is mainly limited by the relatively small number of individuals with primary HIV infection and low number of patients with samples after 24 months of follow-up. This may result in low level of power for analyses of associations at 24 months. Several comparisons are made, and adjustments for multiple comparisons were not performed which may lead to type I errors. A significance level of *p* < 0.05 was used, but a too restrict significance level may lead to type II errors and failing of detecting a difference. Thus, the results presented should be considered in the context of both the number of comparisons and the a priori hypotheses. Furthermore, the study is descriptive. Thus, conclusions on causality cannot be made.

## Conclusions

In conclusion, CD4+ T cell recovery and immune homeostasis was comparable in individuals with primary HIV infection and individuals with chronic HIV infection presenting for care with CD4+ T cell counts <350 cells/μL. Higher plasma TSLP was found in individuals with primary HIV infection compared to both chronic HIV infection and healthy controls. In primary HIV infection, TSLP was associated with lower thymic output, but TSLP was not associated with CD4+ T cell recovery. In contrast, higher plasma TSLP was associated with lower CD4+ T cell recovery in the LP-nonAD. These findings indicate a possible role of TSLP in immune homeostasis in HIV infection but do not support TSLP to affect immune recovery in primary HIV infection. Further studies in independent cohorts are warranted.

## Abbreviations

cART: Combined antiretroviral therapy
CM: Central memory
EM: Effector memory
EP: Early presenters
HC: Healthy controls
LD: Late differentiated
LP-AD: Late presenters with advanced disease
LP-nonAD: Late presenters without advanced disease
PHI: Primary HIV infection
RTE: Recent thymic emigrants
TSLP: Thymic stromal lymphopoietin
